# Purified Clinoptilolite-Tuff as an Efficient Sorbent for Food-Derived Peanut Allergens

**DOI:** 10.3390/ijms25126510

**Published:** 2024-06-13

**Authors:** Carmen Ranftler, Magdalena Zehentner, Andreas Pengl, Andreas Röhrich, Cornelius Tschegg, Dietmar Nagl

**Affiliations:** GLOCK Health, Science and Research GmbH, Hausfeldstrasse 17, 2232 Deutsch-Wagram, Austria; carmen.ranftler@glock.at (C.R.); magdalena.zehentner@glock.at (M.Z.); andreas.pengl@glock.at (A.P.); andreas.roehrich@glock.at (A.R.); cornelius.tschegg@glock.at (C.T.)

**Keywords:** zeolite, purified clinoptilolite, peanut allergy, gluten, gliadin, gastrointestinal tract, dietary allergies, adsorption, artificial fluids, ELISA (enzyme-linked immunosorbent assay)

## Abstract

The avoidance of allergen intake is crucial for persons affected by peanut allergy; however, the cross-contamination of food is common and leads to unpredictable consequences after the consumption of supposedly “safe” food. The aim of the present study was to eliminate harmful traces of peanut allergens from food using purified clinoptilolite-tuff (PCT)—a specially processed zeolite material. Analyses were performed using a peanut ELISA and a Coomassie blue (Bradford) assay. Mimicking conditions of the human gastrointestinal tract demonstrated a higher efficacy of PCT in the intestine (pH 6.8) than in the stomach (pH 1.5). Adsorption rates were fast (<2 min) and indicated high capacities (23 µg and 40 µg per 1 mg of PCT at pH 1.5 and pH 6.8, respectively). Allergenically relevant peanut protein concentrations were sorbed in artificial fluids (32 µg/mL by 4 mg/mL of PCT at pH 1.5 and 80.8 µg/mL by 0.25 mg/mL of PCT at pH 6.8) when imitating a daily dose of 2 g of PCT in an average stomach volume of 500 mL. Experiments focusing on the bioavailability of peanut protein attached to PCT revealed sustained sorption at pH 1.5 and only minor desorption at pH 6.8. Accompanied by gluten, peanut proteins showed competing binding characteristics with PCT. This study therefore demonstrates the potential of PCT in binding relevant quantities of peanut allergens during the digestion of peanut-contaminated food.

## 1. Introduction

### 1.1. Allergens Derived from Food

Food allergies have shown increasing prevalence and incidence worldwide in recent decades. Such allergies affect the quality of life of the person concerned, and their whole family may end up socially and financially affected. Interestingly, teenagers and young adults are more frequently affected by food allergies [1,2].

While sensitization is defined as a positive skin or blood test regardless of the clinical reaction, allergy is specified by a positive allergy test plus signs and symptoms. Food allergies can be verified by skin prick testing (in vivo) or laboratory testing (in vitro) for immunoglobulin E (IgE) in serum, or they can be diagnosed by reaction(s) that are in accordance with the criteria for type 1 hypersensitivity (including cutaneous, respiratory, gastrointestinal, cardiovascular, and central nervous system signs and symptoms) [3]. In regard to IgE-mediated food allergies, approximately 8.3% of children and 10% of adults were affected in the USA in 2018 [1].

To date, the recommendation for preventing food allergies has been to introduce minimal amounts of potential allergenic foods to an individual’s diet at the time of weaning, as there is no efficient cure for existing food allergies other than avoidance. Because of this avoidance, a lack of nutrients is not uncommon in affected patients. Strategies for desensitizing the immune system do not have good outcomes over time; sustained unresponsiveness is not achieved by at least half of patients, and up to 70% of successfully desensitized individuals lose their tolerance after a short period of avoidance. In the late stages of desensitization, when 75% of patients consume peanuts daily or 25% less frequently, allergic reactions are still noted [2].

#### 1.1.1. Peanut (*Arachis hypogaea*)

The prevalence of peanut allergy among children multiplied by 3.5 from 0.4% to 1.4% between 1997 and 2008, while the prevalence of tree nut allergies rose from 0.2% to 1.1% at the same time. Between the first two years of infancy, an Irish birth cohort reported a 1.8% prevalence of peanut allergy in 2016, while a cross-sectional pediatric US food survey examining 3- to 5-year-old children reported a 2.1% prevalence in 2018 [1].

Moreover, there is evidence indicating that approximately 40% of children with food allergies suffer from more than one allergy [2].

Published data show that only 20% of peanut allergies resolve naturally, while 80% are lifelong [1,3]. Furthermore, peanut allergies, together with tree nut, shellfish, and finfish allergies, are more likely to cause severe physical reactions in both children and adults. Food allergies in general cause continuously increasing rates of anaphylaxis (FAIR Health described an increment of 377% from 2007 to 2016), hence increasing emergency department visits and hospitalization rates [1].

Cross-reactions caused by various foods and environmental allergens are known, as homologous proteins are conserved among animals and plants. In the case of whole peanut protein, there is a cross-reaction with proteins derived from the pollens of grass and trees, as well as other seeds, beans, and nuts [3,4]. There are five specific proteins (Ara h 1, 2, 3, 8, and 9) that cause peanut allergy. The positive test results and symptoms of over 95% of patients are attributable to the heat-resistant plant storage proteins Ara h 1, 2, and 3. The Ara h 8 protein is heat-labile and responsible for cross-reactions with pollens. Ara h 8 causes mostly mild reactions, as only 18% of patients with an allergy to this protein alone show symptoms. In southern Europe, Ara h 9 is known to cause severe health problems like those caused by Ara h 1, 2, and 3 [3]. In the literature, it has been indicated that seed proteins not only possess storage functions but also play a role in the defense and resistance systems of plants and interact with pests and pathogens, as there are insects, nematodes, fungi, and viruses deriving from the soil but that can also be air- and foliar borne. These proteins are likewise responsible for the production of specific IgEs when they come into contact with the immune systems of predisposed individuals. Peanut seeds contain many proteins that simultaneously belong to the plant pathogen defense system and cause allergic reactions in humans. The hardly digestible, highly allergenic Ara h 2 and Ara h 6 proteins belong—together with Ara h 7—to the group of 2S albumins; known to have activity against mold and phytopathogenic fungi, they can permeabilize yeast cells and display action against bacteria. Nearly all other known Ara h proteins also have functions relating to pathogen defense. To sum up, peanut allergenic proteins can act on cell membranes, interact with innate immune receptors, and modulate signaling pathways. Concomitantly, there is another reason for the toxic potential of Ara h proteins—their structural similarity to the potential allergenic proteins of other organisms. To stick with Ara h 2, Ara h 6, and Ara h 7, they have homologues in the seeds of dandelions, passion fruits, sunflowers, sesame, and castor beans, and, together with Ara h 9, Ara h 16, and Ara h 17, they belong to the superfamily of prolamins [4]. Other well-known members of the prolamin family are the storage proteins of wheat, barley, rye, oat, corn, rice, and sorghum, named gliadin, hordein, secalin, avenin, zein, oryzin, and kafirin, respectively (Table 1). The first three in particular exhibit highly allergenic potential, hence causing gluten intolerance in sensitive persons. Homologues can lead to health problems, as the body cannot distinguish their native sources and structural twins, leading to cross-allergies, even across species. Therefore, specific symptoms may arise in patients even without their consumption of the particular allergen that they are (known to be) allergic to.

The treatment for a type I hypersensitivity reaction caused by peanuts is injectable epinephrine, which, after intramuscular administration, takes 8 min to show its full effect and reverses more than 90% of the systemic reactions caused by peanut exposure [1,2,3].

#### 1.1.2. Gluten

It is estimated that 10% to 25% of patients with food allergies are affected by an allergy to wheat, which is estimated to be prevalent in 0.33–1.17% of the population worldwide. This allergy consists of an IgE-mediated immune response to various wheat proteins, including gluten proteins [5]. Gluten proteins can be found as equivalents in various crops (especially in barley and rye) and are composed of prolamins (containing ≈15% proline) and glutelins (containing ≈35% glutamine). The amino acids proline and glutamine are relatively resistant to digestion by gastrointestinal proteases, and thus their residues cause an immune response when touching the mucosa in genetically predisposed patients. Potentially toxic gliadin peptides can be found in gluten at a proportion of approximately 10% [5,6].

In addition to gluten-dependent allergy, other gluten-related disorders have been described, such as celiac disease and nonceliac gluten sensitivity [5,7]. All these enteropathies have the following in common: There is no cure, but there is one standard therapy, which consists of strictly avoiding gluten. As a consequence, patients exhibit nutritional deficiencies deriving from the low vitamin and mineral levels of a gluten-free diet, even leading to possible growth retardation in children [7].

Even the consumption of originally gluten-free products can pose a risk due to cross-contamination during harvesting, production, processing, packing, and storage [8]. Because of this problem, the International Codex Alimentarius [9] and, subsequently, EU Regulation 828/2014 [10] specified the content and mandated the correct declaration of gluten present in foods. Only 20 ppm or below is defined as “gluten-free” and thus non-toxic for patients exhibiting celiac disease [9,11]. Unfortunately, there is no such regulation for peanut content in food.

**Table 1:** Peanut [4] and gluten [5,7,12,13] proteins (allergens) and their classification. Gluten consists of a mixture of proteins combining prolamins and glutelins. Their respective names are given to different crops.

Peanut (*Arachis hypogaea*) Protein	Superfamily	Type of Protein
Ara h 1	cupins	vicilin
Ara h 2	prolamins	2S albumin
Ara h 3	cupins	legumin
Ara h 4	cupins	legumin (isoform of Ara h 3)
Ara h 5	profilins	
Ara h 6	prolamins	2S albumin
Ara h 7	prolamins	2S albumin
Ara h 8	plant pathogenesis-related proteins PR-10	
Ara h 9	prolamins	nonspecific lipid transfer proteins
Ara h 10	oleosins	
Ara h 11	oleosin	
Ara h 12	defensins	
Ara h 13	defensins	
Ara h 14	oleosin	
Ara h 15	oleosin	
Ara h 16	prolamins	nonspecific lipid transfer proteins
Ara h 17	prolamins	nonspecific lipid transfer proteins
**Crop** (*Genus*)	**Superfamily**	**Type of Protein**
wheat (*Triticum aestivum*)	prolamins	gliadin
barley (*Hordeum vulgare*)	prolamins	hordein
rye (*Secale cereale*)	prolamins	secalin
oat (*Avena sativa*)	prolamins	avenin
corn (*Zea mays*)	prolamins	zein
rice (*Oryza sativa*)	prolamins	oryzin
sorghum (*Sorghum bicolor*)	prolamins	kafirin
wheat (*Triticum aestivum*)	glutelins	glutenin
barley (*Hordeum vulgare*)	glutelins	hordenin
rye (*Secale cereale*)	glutelins	secalinin
oat (*Avena sativa*)	glutelins	avelanin
corn (*Zea mays*)	glutelins	zeanin
rice (*Oryza sativa*)	glutelins	oryzinin
sorghum (*Sorghum bicolor*)	glutelins	-

### 1.2. Zeolite—Structure, Application, and Purification

#### 1.2.1. General Information concerning Structure and Application of Zeolites

Zeolites are minerals of either natural or synthetic origin. Naturally occurring zeolites were discovered almost 270 years ago by Baron Axel Frederick Cronstedt in 1756. Since then, they have been well studied chemically, physically, and mineralogically, so not only a wide spectrum of applications but also different newly created forms with specific characteristics exist today [14]. Over 200 distinct types of zeolites are known, and, among them, more than 40 are naturally formed [15].

Natural zeolite-tuff deposits can be found all over the world, as they occur in various geological settings formed during different geological eras; their volume, mineralogical composition, and quality, however, greatly vary [16,17]. Zeolite is formed through volcanic eruptions, when ash (glass particles) makes contact with (saline) water, thereby forming zeolite [16].

Zeolites not only store and release water but are also able to act as molecular sieves by replacing the atoms within their crystal lattices without losing their molecular structure [14,15]. Due to their ion exchange capacity, their applications are diverse in industrial and agricultural fields: they can be used in building materials and cements; for soil amendments; to bind radioactive ions such as cesium and strontium, as well as heavy metals; as cleaners for (waste-) water and gases; as kitty litter; etc. Moreover, zeolites have biological and medical applications, including in dietary supplements, cosmetics, and wound healing [16,18,19,20].

#### 1.2.2. Clinoptilolite and Purified Clinoptilolite-Tuff

Clinoptilolite is a natural member of the zeolite mineral family. As another representative zeolite, it consists of linked SiO_4_ tetrahedrons, which form a porous network of channels and cages [17,21]. The partial substitution of Si^4+^ by Al^3+^ leads to an overall negatively charged crystal lattice, mainly neutralized by exchangeable extra-framework cations. This combination of a porous structure with a negatively charged framework and exchangeable ions is responsible for this mineral’s ion exchange and sorption capacities [17,21]. Clinoptilolite itself is of the heulandite type. The raw material used in this study originates from an open-pit mine in Nižný Hrabovec in the eastern Slovak Republic, and its exact chemical formula is (Ca_1.51_K_1.39_Mg_0.37_Na_0.15_) [Al_5.64_Si_26.36_O_72_] ∙ 11.77 H_2_O [21]. Being of volcanic origin, clinoptilolite consists of elements undesirable for human consumption and application. Although the quality of the clinoptilolite-tuff in Nižný Hrabovec is high compared to that of other deposits, it is still necessary to purify the raw material according to a fully quality-controlled and patented process involving ion exchange, micronization, and terminal heating. The resulting purified clinoptilolite-tuff is safe for consumption and marketed in the USA as G-PUR^®^ [17,21,22].

#### 1.2.3. Purified Clinoptilolite-Tuff as Sorbent of Different Toxic and Harmful Substances

Zeolites are excellent sorbents (adsorbers/absorbers) of different types of substances [16]. Recent human studies involving PCT revealed its capacity to bind various elements and molecules, with a good tolerability profile in humans when applied both externally and internally [18,20,23,24]. The following health-relevant heavy metals were sorbed to PCT: nickel, cadmium, cesium, barium, thallium, and lead in vitro [19]; in addition, lead was also used in human studies [18,24]. Moreover, in vitro experiments demonstrated the binding of miscellaneous organic material to PCT: *Clostridium difficile* toxins A and B [25], gluten [26], and different coronavirus family members, including SARS-CoV-2 [27].

In particular, the adsorption of gluten from at least 80 mg to a daily intake dose of 2 g on PCT raised the question of whether peanuts—another source of allergen—could also be neutralized by PCT. As peanut allergies are quite common, with their prevalence rising and no cure being available except for intake avoidance, the adsorption of any quantity of peanut allergens present through the cross-contamination of originally non-peanut-containing food could help to improve patient wellbeing. Some peanut allergens belong to the superfamily of prolamins, which are a major component of gluten. In a previous study, these were successfully subjected to PCT binding [26].

The aim of the work presented in this publication was to determine whether peanut allergens can bind to PCT and thereby lose their allergenic potential, particularly under conditions mimicking physiological processes. Here, we demonstrate the binding of peanut allergens to purified clinoptilolite-tuff at a pH typical of the stomach (pH 1.5) and intestine (pH 6.8) in buffers as well as in artificial gastric and intestine fluids. Values of up to 23 µg and 40 µg of protein per 1 mg of PCT were documented for pH 1.5 and pH 6.8, respectively. Adsorption took place within the very first minutes under all conditions tested. Experiments conducted in artificial fluids revealed that, once bound, peanut components are nearly irreversibly adsorbed to PCT, and desorption widely fails. Interestingly, competing binding studies with gluten and peanut suggest equal binding sites of both allergens to PCT. Taken together, the binding of certain amounts of peanut allergens provides help to affected persons in overcoming the difficulties caused by peanut-cross-contaminated food.

## 2. Results

### 2.1. Adsorption of Peanut Protein on Purified Clinoptilolite-Tuff

The primary question was as follows: does PCT bind peanut protein in buffers and, if so, in what amount? Subsequent Experiments in artificial gastric and intestinal fluids should provide a more detailed insight into the practical use of PCT for humans with peanut protein allergies.

#### 2.1.1. The Kinetics of Peanut Protein Adsorption to PCT at Different pH Values in a Concentration-Dependent Manner

**Figure 1:**
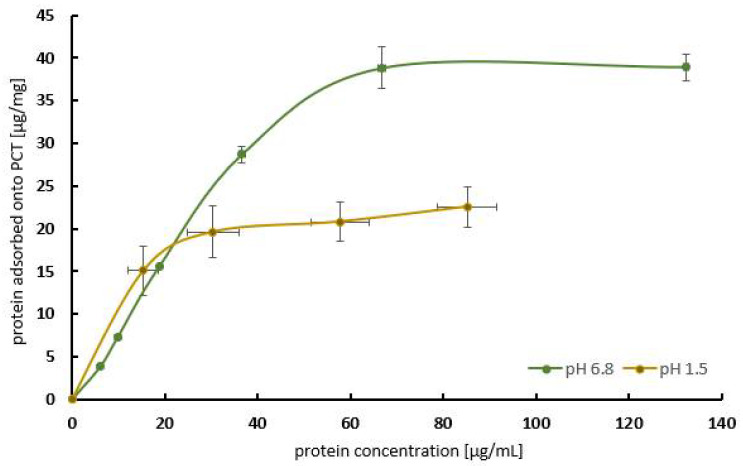
Saturation curves. Amount of bound peanut protein with increasing peanut protein concentration at pH 6.8 (green line) and pH 1.5 (brown line). Saturation at 37 °C was reached with adsorption capacities of 40 µg/mg (pH 6.8) and 20 µg/mg (pH 1.5). The content of peanut protein was determined using a Coomassie assay (Bradford assay). Each curve represents the data of two consecutive experiments carried out in quadruplicate. Standard deviations are depicted for each peanut protein concentration used.

**Figure 2:**
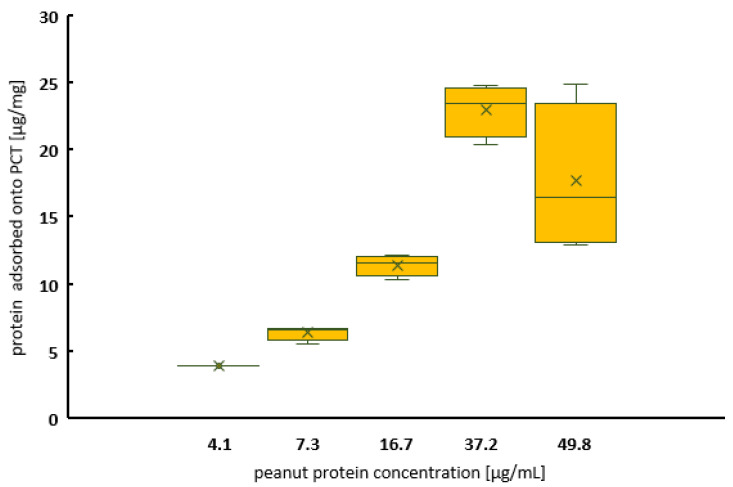
Boxplot—saturation at pH 1.5, determined using ELISA. Adsorbed amounts of up to 23 µg of peanut protein per 1 mg of PCT quantitatively confirm the previous results obtained with Coomassie assay (Bradford assay). The x in each box marks the arithmetic average value. The graph represents the data from two consecutive experiments, each carried out in quadruplicate.

The first experiments were performed to obtain a saturation curve at two different pH values in order to imitate the nearly neutral level of the intestine (pH 6.8, Figure 1) and the highly acidic level of the stomach (pH 1.5, Figure 1 and Figure 2) at 37 °C. Therefore, 1 mg/mL of PCT was incubated with various concentrations of peanut protein (up to 140 µg) extracted from peanut powder for an analysis of both at pH 6.8 and at pH 1.5. The analyses were performed by using a Coomassie assay, and, for the gastric pH conditions, they were confirmed using an ELISA [28,29]. The buffers were blended corresponding to the test solutions of the European Pharmacopoeia [30].

Figure 1 characterizes the adsorption properties of PCT for peanut protein with an increasing allergen concentration in the test solution without further interference factors at two different pH values. A maximum of approximately 40 µg of peanut protein per 1 mg of PCT was bound at a nearly neutral pH (pH 6.8). This means that about 80 mg of peanut protein would be adsorbed by a total daily intake of 2 g of PCT. Saturation was reached at protein concentrations above 60 mg/mL in the medium (Figure 1, green curve). A similar picture was obtained under acidic conditions (pH 1.5). However, due to the lower adsorption capacity of about 23 µg/mg at pH 1.5, saturation was already reached at an initial protein concentration of 30 µg/mL. Although the binding of peanut protein to PCT at pH 1.5 was lower, it was still high enough to be relevant (Figure 1, brown curve). The adsorption characteristics at pH 1.5 were confirmed using an ELISA measurement (Figure 2).

**Figure 3:**
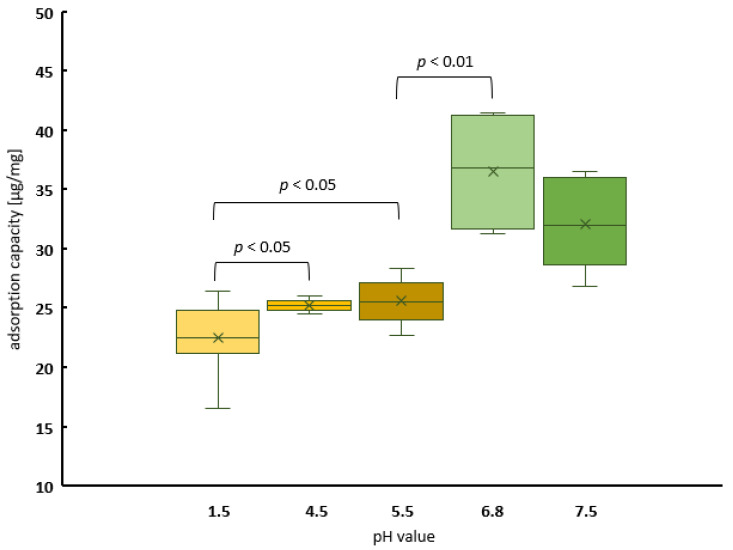
Boxplot—pH-dependent adsorption of peanut protein onto PCT. Adsorption capacities at almost neutral pH (6.8) are highly significant and better than those determined in acidic matrices (from pH 1.5 to pH 5.5). The lowest amount of bound protein (approximately 22 µg/mg) was calculated for stomach pH. The difference in the average values at pH 1.5 and pH 4.5 was still statistically significant. Protein concentrations after incubation at various pH levels were determined using Coomassie assay (Bradford assay). The x in each box marks the arithmetic average value. The graph represents the data from two consecutive experiments, each carried out in quadruplicate.

Because of the results obtained, a more detailed investigation of the pH dependency of the adsorption capacity was carried out. Once more, the recommended test solutions of the European Pharmacopoeia [30] were utilized for this purpose, and peanut powder again served as the source of peanut protein (Figure 3). The maximum amount of bound analyte was determined under conditions of saturation (i.e., peanut protein available in excess). As before, at low pH levels, the binding of the peanut protein to PCT was lower than that at higher levels. Up to 40 µg of protein per 1 mg of PCT could be adsorbed at neutral pH, with just more than half of this (22 µg/mg) adsorbed at pH 1.5.

#### 2.1.2. The Kinetics of Peanut Protein Adsorption to PCT in a Time-Dependent Manner

The next step was to analyze the speed of the reaction at nearly neutral pH. For this reason, the calculated 40 µg (finally achieving 35 µg) of peanut protein extracted from raw peanut mush was incubated with 2 mg of PCT from 30 min to 0 min, reduced in a buffer with a pH of 6.8, and subsequently analyzed using an ELISA.

**Figure 4:**
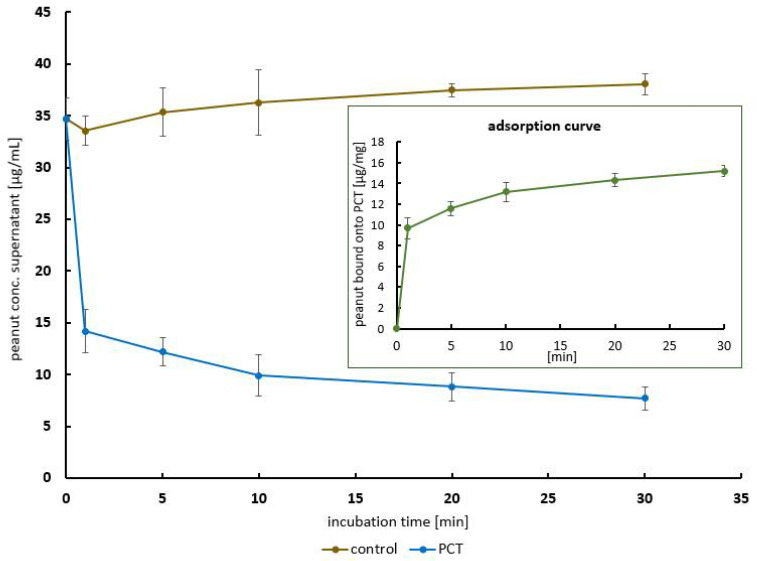
Kinetic curve—adsorbed amount of peanut protein during the first 30 min of incubation. The brown line refers to the amount of protein in the control, while the blue line shows the decline in the supernatants in a time-dependent manner. Peanut protein was quantified using ELISA. In addition, the corresponding (calculated) amount of bound peanut protein is given in the inserted diagram (green line). Approximately two-thirds of the reaction was completed within the first few minutes of incubation. At this point, 10 µg of peanut/mg PCT was bound. Afterwards, the binding rates decreased with an increasing incubation period. The adsorption capacity in this experimental setup reached 16 µg/mg. The graph represents the data from three consecutive experiments each carried out in quadruplicate. Standard deviations are depicted for each protein concentration used.

As depicted in Figure 4, adsorption occurred rapidly. Within the very first minutes, the highest proportion of the protein was bound to PCT. Adsorption continued but with an increasing degree of saturation, and the binding velocity decreased. In the experiment shown, the adsorption capacity adds up to 15 µg peanut protein per 1 mg of PCT.

### 2.2. Neutralization of Peanut Protein by Purified Clinoptilolite-Tuff in an Amount Relevant for Allergic Persons

#### 2.2.1. The Adsorption of an Allergy-Relevant Concentration on PCT in Test Solution at pH 6.8

**Figure 5:**
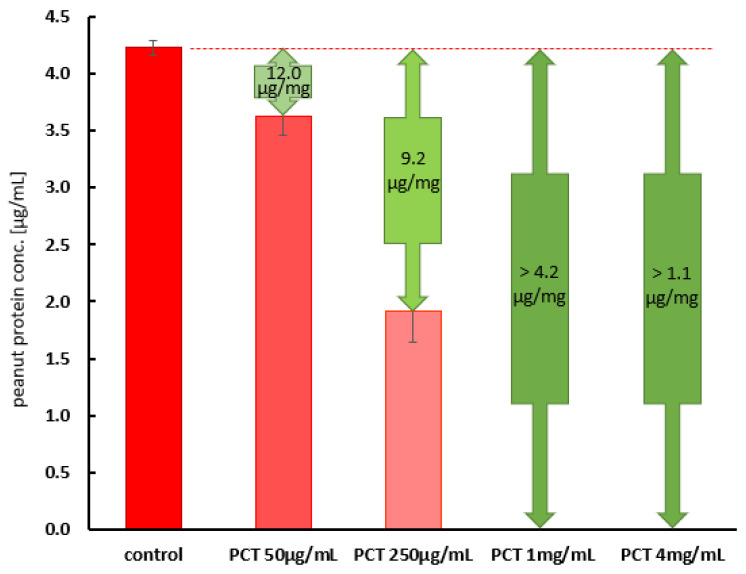
Adsorption of a relevant amount of peanut by different doses of PCT at pH 6.8. Protein solutions were incubated with increasing concentrations of PCT at 37 °C and subsequently measured using ELISA. The green arrows display the calculated quantity of bound protein per 1 mg of PCT for each condition. PCT concentrations far below 4 mg/mL (25% of a daily dose of 2 g suspended in 500 mL gastric fluid) are sufficient to neutralize peanut protein concentrations of 4 µg/mL (corresponding to a peanut protein intake of 2 mg in the same stomach volume). The graph represents the data from two consecutive experiments, each carried out in quadruplicate. Variations in color illustrate the decreasing free peanut protein (red) and increasing PCT concentration (green), respectively.

The next step was to analyze the relevance of the reaction. For this reason, 4 µg/mL of peanut protein was applied and analyzed using an ELISA. This concentration theoretically corresponds to 2 mg of peanut protein in a stomach volume of 500 mL. Hence, a concentration of 4 mg/mL of PCT corresponds to a single dose of 2 g of PCT per day in the same stomach volume. As shown in Figure 5, only one-quarter of the daily intake was sufficient to neutralize the entire amount of peanut protein added to the buffer solution (pH 6.8).

#### 2.2.2. The Adsorption of Allergy-Relevant Concentrations on PCT in Artificial Gastric and Intestinal Fluids

To characterize the adsorption process in a more realistic setup, testing was performed in artificial gastric and intestinal fluids. To maintain the functionality of the ELISA antibodies, the enzymes pepsin and pancreatin were deactivated thermally prior to the mixing of the artificial fluids.

**Figure 6:**
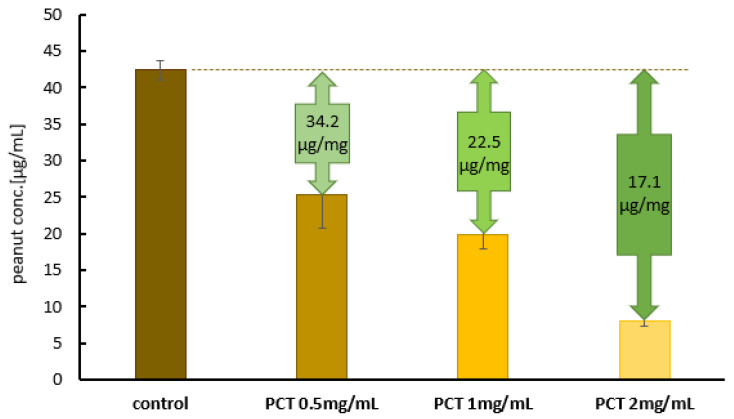
Adsorption of peanut allergen (derived from peanut mush) onto PCT in artificial gastric fluid (agf). Peanut mush was suspended in agf and incubated at 37 °C without (control) and with PCT at different concentrations. Supernatants were analyzed using peanut ELISA. The green arrows display the calculated quantity of bound peanut material per 1 mg PCT for each condition. The initial peanut concentration above 40 µg/mL was reduced to 8 µg/mL by PCT at a concentration of 2 mg/mL. A representative result is shown. Variations in color illustrate the decreasing free whole peanut (brown to yellow) and increasing PCT concentration (green), respectively.

Figure 6 illustrates the amount of bound whole peanut in the artificial gastric fluid at different PCT concentrations. The initial peanut concentration in this experimental setup was chosen to be approximately 40 µg/mL (control), high enough to ensure complete saturation at all tested PCT concentrations but still quantify peanut levels in the supernatants after incubation. A PCT concentration of 4 mg/mL, corresponding to a daily dose of 2 g suspended in 500 mL of gastric fluid (stomach contents), was enough to bind the vast majority of whole peanut. Under these conditions, only 8 µg/mL of peanut material remained in the supernatants (rightmost bar). With half the PCT concentration (2 mg/mL), more than half of the available peanut components was adsorbed. At the lowest concentration tested (1 mg/mL), 17 µg of peanut was bound to PCT. The calculated amounts of bound peanut material per 1 mg of PCT for the different conditions (approaches) are given in the green arrows above the bars in Figure 6.

Because of the previously observed higher adsorption capacity at neutral pH, the initial peanut concentration in the tests with the artificial intestinal fluid was chosen to be 100 µg/mL. As the whole peanut could not be detected after incubation with 4 and 2 mg of PCT/mL, the PCT concentration was gradually reduced until peanut compounds could be quantified in the supernatant. Therefore, it could be assumed that PCT was saturated under these conditions (0.25 mg of PCT/mL).

**Figure 7:**
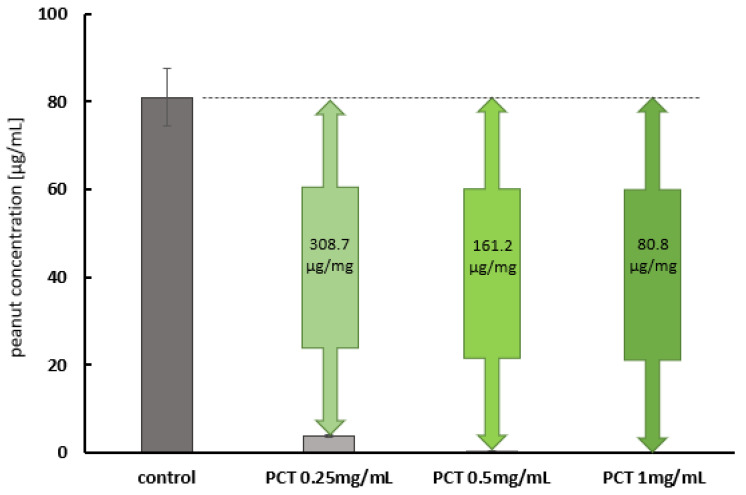
Adsorption of peanut allergen (derived from peanut mush) onto PCT in artificial intestinal fluid (aif). Peanut mush was suspended in aif and incubated at 37 °C without (control) and with PCT at different concentrations. Quantification of the peanut allergen in the supernatants was performed using peanut ELISA. The green arrows display the calculated quantity of bound peanut material per 1 mg PCT for each condition. Corresponding to the much higher adsorption capacity at neutral pH, a PCT concentration of 0.5 mg/mL was high enough to completely neutralize a peanut concentration of 80 µg/mL. A representative result is shown. Variations in color illustrate the decreasing free whole peanut (grey) and increasing PCT concentration (green), respectively.

Figure 7 presents the corresponding adsorption data generated in the matrix of the artificial intestinal fluid. In fact, the adsorption of peanut protein onto PCT seems to be more efficient in the artificial intestinal fluid than in the artificial gastric fluid. On the one hand, this confirms the tests on pH dependency; on the other hand, it is surprising, especially in view of the high protein content of the intestinal fluid (i.e., 10 mg of deactivated pancreatin per mL). These results might be promising in terms of achieving an effect in the human intestine.

### 2.3. Desorption of Peanut Protein from Purified Clinoptilolite-Tuff

**Figure 8:**
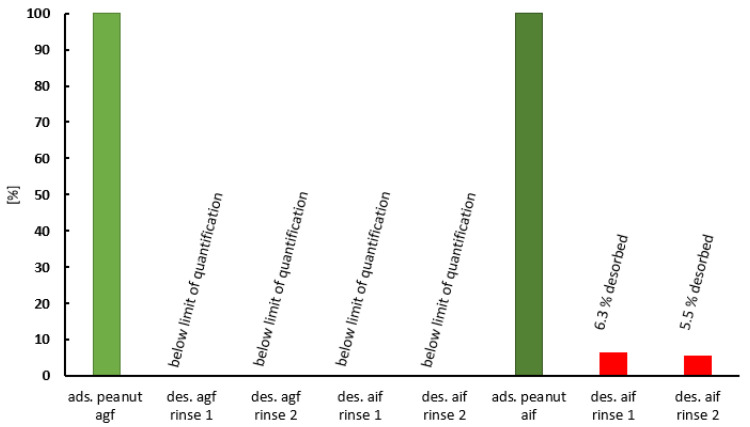
Desorption of previously boundpeanut compounds from PCT. PCT was saturated with peanut allergen (derived from peanut mush) in a matrix of artificial gastric (agf) or intestinal juice (aif). In the next step, it was rinsed twice with either artificial gastric or intestinal fluid. The irrigation fluids (rinse 1 and rinse 2) were analyzed for traces of peanut allergen using ELISA. The amount of adsorbed (ads.) peanut compounds is displayed in green color, any desorbed (des.) peanut allergens are highlighted in red. Once under gastric conditions, adsorbed peanut compounds were not desorbed any more by either agf or aif. In aif, adsorbed peanut was eluted from PCT to a low extent. The graph represents the data from two consecutive experiments, each carried out in quadruplicates.

At this point, it should be clarified whether bound peanut allergen could be desorbed from PCT and, if so, to what extent. Again, experiments were performed with different concentrations for the artificial gastric and intestinal fluids. For this purpose, PCT was saturated with peanut allergen in artificial gastric or intestinal fluid and then separated via centrifugation, as described in the Section 4. In the second step, the acquired pellet consisting of peanut protein bound to PCT was resuspended in fresh fluid (either gastric or intestinal), incubated, and separated again. The resulting supernatants were subsequently analyzed for traces of peanuts. Impressively, Figure 8 shows that the peanut components, once adsorbed onto PCT in the gastric environment, were not desorbed under artificial gastrointestinal conditions. When rinsed in the artificial intestinal fluid, however, the adsorbed peanut content resuspended to a very small extent. This might be due to the particularly high amount of bound protein on the saturated PCT.

The results indicate that the largest proportion of adsorbed peanut allergen was no longer bioavailable after adsorption, as it could not be desorbed from the PCT under the tested conditions.

### 2.4. Competing Adsorption of Peanut Protein and Gliadin onto Purified Clinoptilolite-Tuff

**Figure 9:**
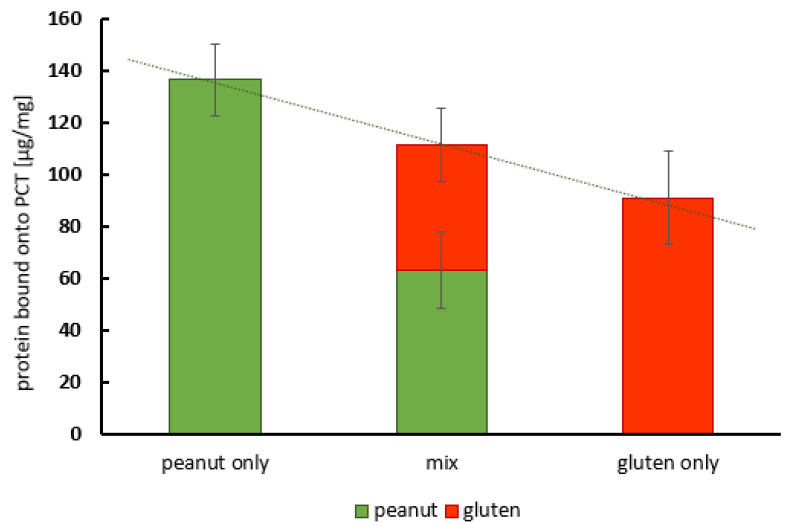
Adsorption of peanut protein and gluten onto PCT, showing either peanut- or gluten-only ELISA results or those obtained under competing conditions (combined use of allergens is depicted by “mix”). The simultaneous use of both allergens in “mix” did not lead to an increase in overall loading capacity of PCT as shown by the dotted line. Both types of protein compete for the same binding sites. Even the ratio of peanut protein to gluten remained nearly the same. The graph represents the data from three consecutive experiments, each carried out in quadruplicate.

In addition to the previous experiments, it was of special interest to ascertain whether the adsorption characteristics differed from those observed with gluten allergen [26]. To this end, PCT was incubated with almost equal concentrations of peanut protein or gluten, as well as with peanut protein and gluten mixed in an equal ratio. The adsorbed amounts of allergen in these test approaches were analyzed using either a peanut or gluten ELISA [31] and calculated (see Table 2 for the values from one representative experiment). They are illustrated in Figure 9.

The left (green) and right (red) bars in Figure 9 show the amounts of peanut protein and gluten adsorbed onto PCT, respectively, under conditions with one allergen only (peanut protein or gluten). Although both analytes were used at approximately the same concentration, a slightly higher adsorption capacity was calculated for peanut protein (approximately 135 µg/mg) than for gluten (90 µg/mg).

The bar in the center reflects the adsorbed amounts of both allergens under competing conditions with peanut protein (green) and gluten (red) in the same test samples. In total, 110 µg of allergen was adsorbed onto 1 mg of PCT, which means that the overall adsorption capacity remained the same. This clearly shows that peanut protein and gluten compete for the same binding sites on PCT.

Under these conditions, the ratio of PCT-bound peanut protein (63 µg) to gluten (41 µg) remained nearly the same as that in the experimental approaches with only one of the two analytes. This indicates that neither of them preferentially adsorbed to the mineral (PCT).

It can be assumed that both allergens bound with nearly equal affinity to the same binding sites of PCT.

## 3. Discussion

The prevalence of food allergies in general and peanut allergy in particular is rapidly increasing [1]. “May contain traces of peanuts” is optional information provided by food producers called PAL (precautionary allergen information labeling), and it serves to provide a warning of an imminent risk, concomitantly reducing the number of available products for allergic consumers—sometimes as a simple precautionary measure [32]. There is no uniform legislation defining a specific limit for the declaration of peanut traces, and the regulation of the European Union requires only information on the intended ingredients [33].

In a previous study, peanut protein was found to be highly bioavailable in in vitro digestion experiments: when measuring raw peanut material, the value was nearly 93%, and, when processed, it still reached 81%, which was higher than that for soy, amounting to 72% [34].

The eliciting dose (ED) for a peanut allergic reaction in 5% (≙ ED05) of the population with peanut allergy was found to be 1.5 mg of peanut protein, which corresponds to 6 mg of whole peanut, and the ED for 1% (≙ ED01) was defined as 0.2 mg [35,36]. Half of the population with peanut allergy (corresponding to ED50) displayed an allergic reaction after exposure to 3 mg of peanut protein. The no-observed-adverse-effect level (NOAEL) was described as 30 µg of peanut protein; below and up to this level, consumption is considered to be safe for people affected by peanut allergy. Generally, patients with severe symptoms react to lower doses than those affecting patients with mild symptoms [37]. In the experiments performed in the present study (Figure 5), peanut protein was used at a concentration of approximately 4 µg/mL, corresponding to 2 mg in a 500 mL stomach volume. Simultaneously, 4 mg/mL of PCT, corresponding to 2 g in a 500 mL stomach volume (a daily single dose), was used to bind the peanut protein. It was shown that less than 1 mg/mL of PCT was sufficient to eliminate the entire amount of peanut protein from the buffer solution at nearly neutral pH. Hence, the complete dose of PCT (2 g) is capable of binding peanut protein concentrations far above ED50 (8.4 mg of peanut protein per 2 g of PCT). Kinetic studies performed at pH 6.8 revealed a high velocity of the reaction: Within the first 2 min, more than 20 µg/mL of the 35 µg/mL concentration of peanut protein was adsorbed, which corresponds to nearly two-thirds of the sorption capabilities in this experimental setup. The adsorption capacity reached 15 µg of peanut protein per 1 mg of PCT (Figure 4). This phenomenon of rapid adsorption has not only been demonstrated for peanut proteins but also for *Clostridium* toxins [25], gluten [26], and even other groups of substances such as aflatoxins [38]. Obviously, these substances all have the following in common: they are characterized by a high affinity for the surface of clinoptilolite. Key factors in this regard are, for example, local differences in the electrical charge and/or hydrophobicity of the molecules. The extent to which each factor plays a role has not yet been clarified in detail. The fact is that aflatoxins, known as hydrophobic molecules, bind very efficiently onto clinoptilolite. Additionally, gliadins (the allergenic fraction of gluten), which are more soluble in alcohol (70% *v*/*v*) than in water, bind onto PCT very well and rapidly [12,13,39,40,41].

The adsorption showed pH dependency (Figure 3), and adsorption capacities of 40 µg/mg (≙ 80 mg in 500 mL) at pH 6.8 and 20 µg/mg (≙ 40 mg in 500 mL) at pH 1.5 could be defined (Figure 1 and Figure 2). This PCT phenomenon with respect to pH dependency is already known and has been described [19,21,42]. Even minimal changes in pH towards alkalinity produced detectable results, as revealed by the adsorption of heavy metal ions to PCT. Slight amounts of CO_2_ derived from air and accumulated in water (thereby creating carbonic acid), used for analysis, were vaporized during an experimental procedure (warming up). This led to higher standard deviations with respect to cesium, thallium, and lead [19]. When using natural siderophores and clinoptilolite-tuff for sorption experiments, the highest binding capacity of coprogen and iron was detected at pH 4, but it decreased constantly to pH 10, while the most efficient release was observed at pH 8 [42]. Depending on the specific ion or protein, the pH value plays a crucial role in the process of sorption/desorption to/from PCT. The results of a Student’s *t*-test showed a highly significant difference (*p* < 0.01) between the sorption behavior of peanut protein to PCT at a nearly neutral pH of 6.8 and pH 5.5, even though the difference in pH was only 1.3. Moreover, the sorption capacity in the stomach (pH 1.5) was still significantly lower than at pH 4.5 or pH 5.5 (Figure 3).

Traces of peanut proteins may originate not only from food-processing pathways [43] but also structural similarities to evolutionarily conserved proteins of other organisms that are also allergens [4]. In the case of cross-contamination, Krejner-Bienas and colleagues found clinically relevant amounts of peanut allergens in nearly one-third of products declared to potentially contain peanut material [43]. In a trial of randomly chosen bakery products that did not intentionally contain peanuts conducted in the Miami and New York metropolitan areas, it was revealed that 2.6% contained peanut material at levels ranging from 0.1 mg/100 g to 650 mg/100 g, corresponding to an estimated intake of 0.07 mg to 832 mg of peanut protein—amounts capable of triggering a reaction [44].

Another factor causing increasing problems with regard to peanut intolerance is that peanuts are being incorporated more often in diets and other applications; for example, in 2016, 3.3 kg of peanuts were consumed in the USA per capita. In addition to minerals and vitamins, mature peanut seeds contain about 20–25% protein and 45–50% oil [4]. Approximately half of the peanuts produced worldwide are used for oil extraction, as 50 million metric tons are produced per year, and thereof 25 million metric tons of oilseed cake, which is defatted peanut meal containing about 50% peanut protein, are produced as a byproduct. This solid material is traditionally used for animal feed; however, as peanuts contain high levels of proteins, attempts are increasingly being made to utilize them in treating human aliments, which, in turn, is leading to an increase in people coming into contact with peanuts. For example, peanuts can be used as flour in baked goods, including in breads for gluten-sensitive consumers; as plant “milk” in regions in which refrigeration is unavailable; as “cheese”; and as a meat substitute (e.g., tofu-like products). Other uses of peanut protein isolates include gels, emulsifiers, salt substitutes, and bioactive peptides. As several amino acids for optimal human supplementation are present at low levels, transgenic peanut lines have been developed to improve nutritional value. An analysis of the bioavailability of peanut protein has revealed that 92.65% of raw peanut material can be digested in vitro. On the contrary, the protein in roasted peanuts is more stable than that in raw or boiled peanuts. Interestingly, data show that peanut allergenicity is higher in regions where peanuts are consumed roasted (e.g., USA, UK, and Canada) than in parts of the world where peanuts are boiled (e.g., Asia); in the latter regions, the prevalence is reduced, and this has also been confirmed in animal models. Heat treatment can denature peanut protein, thereby reducing but not eliminating its allergenic effect [34]. This also affects the nutritional value of roasted peanut paste by limiting the amino acid sequences of lysine, threonine, and methionine, whereas these three amino acids are equally limited in blanched but unroasted peanut paste [45].

A detailed in vitro analysis of peanut allergens subjected to pepsin digestion mimicking gastric activity revealed distinct differences between the allergens tested. While Ara h 1 and Ara h 3 were hydrolyzed rapidly, Ara h 2 and Ara h 6 showed resistance to even very high concentrations of pepsin; this is in accordance with the reported allergenic effects. Ara h 2 and Ara h 6 in particular have been reported to trigger the immune system in affected persons to a greater extent than Ara h 1 and Ara h 3. Ara h 2 is viewed as the dominant antigen in peanuts and, together with Ara h 6, is the most harmful for persons affected by peanut allergy. Both have a tightly coiled structure and heat stability. In addition to pepsin, Ara h 2 was also exposed to trypsin before the protein fragments resulting from proteolysis were analyzed. It became clear that a range of relatively large 10 kDa peptides that were digestion-resistant were generated and can be considered to pose a potential risk of eliciting allergic reactions—a phenomenon not detected in Ara h 1 and Ara h 3 peanut proteins, which are unstable in a gastric environment and, consequently, quickly broken down into smaller amino acid chains [46,47]. Digestion resistance is often in relation to food allergens [48]. Moreover, polyphenols (such as those from blueberries and cranberries, as well as those from peanut skin) were reported to block the binding sites of the Ara h 1 protein, where the IgE epitope is located, through conformation changes, leading to reduced allergenicity [34].

Another means of mitigating or preventing food allergies is the use of protease inhibitors such as *para*-aminobenzamidine. The idea is that resistance to digestion can prevent proteins from being degraded and, subsequently, the fragments from being adsorbed. Therefore, the induction of an immune response cannot take place, as no allergen fragments bind to IgE, and thus an allergic reaction is not induced. Ara h 1 and Ara h 2 were attached to *para*-aminobenzamidine in in vitro digestion experiments employing the enzyme trypsin. Both allergens were found to be more resistant to degradation, and their allergic capacity was also reduced compared to that of native allergens when bound to *para*-aminobenzamidine [49].

Purified proteins from peanuts are easier to handle in experiments than the whole peanut itself. Although proteins isolated from their “native environment” can change their conformations, as well as their behavior, due to the absence of polysaccharides and/or lipids, they lack protease inhibitors and protein–protein interactions. In particular, when investigating the susceptibility of peanut food allergens to proteolysis, these factors play a crucial role in the bio-accessibility and bioavailability of allergenic epitopes. For this reason, in vitro studies attempt to mimic the conditions of the human gastrointestinal tract with both artificial fluids and the whole peanut matrix. It would be especially interesting to determine which protein fragments are created by or still preserved after digestion and show potential epitopes capable of causing an allergic reaction after contact with the mucosa. Based on these considerations, experiments involving peanut mush (whole peanut matrix) in artificial gastric and intestinal fluids were performed in this study, in addition to those conducted in a pH 6.8 buffer and pH 1.5 test solution. Again, the favored condition for adsorption was pH 6.8 in the artificial intestinal fluid—as it was in the pH 6.8 buffer before. While the calculated quantity of adsorbed whole peanut per 1 mg of PCT was 22.5 µg in the artificial gastric fluid at pH 1.5 (Figure 6), it was found to be 80.8 µg in the intestinal fluid (Figure 7). Using whole protein for experiments is of particular value, as demonstrated when Di Stasio [48] and colleagues confirmed the results reported by Koppelman et al. [47] with regard to Ara h 2 and Ara h 6 being hardly digestible proteins, but, astonishingly, Ara h 3 also showed large-sized fragments of 7–21 kDa when whole peanut and not only an extract was used in experimental testing. This discrepancy can be explained by the effect of the peanut matrix (consisting of lipids and polysaccharides) on single proteins, preventing them from unstoppable degradation (delaying or impairing proteolysis). Even more alarming is the fact that IgE-binding sites were created during proteolytic action. In the case of Ara h 3, the hidden epitope in the native protein was available for interaction with immune system effectors upon gastrointestinal release. Furthermore, Ara h 3 has two more linear epitopes, which are available for IgE binding when the protein still displays its native folding behavior. Conversely, Ara h 1 was completely degraded (2 kDa), and none of the resulting peptides showed immunoreactivity [48]. This contradicts other scientific investigations published, which, irrespective of their degradation by pepsin, describe Ara h 1 fragments as still being toxic when touching the mucosa of the gut. By using a recombinant rAra h 1 form (which was heat-treated, thereby causing structural changes in the protein), Tian et al. [50] showed that heat could reduce the proinflammatory potential of rAra h 1 fragments—an effect also observed in successful pepsin digestion after heat treatment. Experiments were performed with Caco-2 human intestinal epithelial cells, and the activation of IL-8 after the addition of rAra h 1 fragments was analyzed using an ELISA. Interestingly, in more than 90% of peanut-sensitive patients, Ara h 1 could be recognized by serum IgE. The location of epitopes also had a clear effect on the allergenic potential: epitopes in the 393–402 and 498–508 regions of Ara h 1 were recognized by 60% and 90% of patients with peanut allergy, respectively [50]. Our experiments demonstrated the binding of whole peanut to PCT under gastric and intestinal conditions and in the presence of enzymatically inhibited pepsin and pancreatin, respectively. To achieve results in the ELISA, both enzymes were previously inactivated via heat treatment, thus preventing them from acting on the antibodies of the assay. Due to the strong affinity of PCT for the proteins of the peanut matrix, it seems highly probable that large quantities, even after digestion, would be sorbed. Protein fragments bound to PCT would then be excreted without irritating the mucosa. However, further experiments are needed to verify this hypothesis. To ensure that the sorption of peanut allergens to PCT is stable during the artificial digestion process, desorption experiments were performed using a two-step process: adsorption of the peanut compounds to PCT in artificial gastric or intestinal fluid to the point of saturation with the peanut matrix, followed by desorption in either gastric or intestinal fluid. The same pattern was observed in previous experiments: the level of binding at a highly acidic pH was less effective than at nearly neutral pH; however, there was no detectable desorption after rinsing with artificial gastric or intestinal fluid. The whole peanut material was irreversibly bound to PCT. In the case of adsorption in the artificial intestinal fluid, desorption resulted in a small amount of approximately 5.5–6.5% of previously bound peanut material in the fluid. Although higher amounts of whole peanut were adsorbed, the portion released was relatively small (Figure 8).

Previous studies have revealed that, soon after the beginning of oral immunotherapy, basophil sensitivity decreases and levels of allergen-specific, circulating IgG, IgG4, and IgA antibodies increase in patients with IgE-mediated food allergy only with sustained and not transient clinical responses. The clinical efficiency of oral immunotherapy is characterized by functional suppression and not the levels of allergen-specific antibodies. A recent study analyzing blood samples from patients who previously underwent peanut oral immunotherapy with sustained and transient responses revealed unique tolerance-associated conformational epitopes of Ara h 2, recognized by the distinct allergen-specific paratopes of neutralizing IgG antibodies and the subsequent blocking of the IgE-mediated immune reaction. This particular induction of unique Ara-h-2-specific neutralization antibodies was a critical factor that promoted the durability of allergen tolerance [51]. PCT probably helps transient responders who do not develop highly specific antibodies after oral immunotherapy and show severe allergic symptoms by reducing the number of peanut allergens after ingestion to a level uncritical for them.

Since 2020, a new specific immunotherapy for the hypo-sensitization of peanut allergy has been available on the market (AR101, trade name Palforzia). The aim of this is to diminish the allergic reaction occurring after peanut consumption. Increasing amounts of peanut material are orally administered over several weeks, resulting in a habituation effect on the body and hence attenuation of the immune response. Studies have revealed the possibility that treatment could result in higher dose tolerance and lower symptom severity. Additionally, the discontinuation of or a reduction in the therapy increases the likelihood of relapse and becoming clinically reactive to peanuts once again. Moreover, the therapy bears a risk of anaphylaxis and some extensive cofactor restrictions, as well as the possibility of lifelong administration [52,53]. PCT has already been described to be safe in long-term applications [18].

Additionally, peanuts are histamine liberators. Diamine oxidase (DAO) was described as not being a very reliable marker for histamine intolerance in a large Swedish random-population-based study, as there were too many influencing factors, such as BMI (body mass index) and age in a sex-dependent manner [54]. Selvam and coworkers verified the binding capacity of zeolite with respect to histamine in in vitro experiments [55,56]. PCT is a processed clinoptilolite-tuff, with levels of heavy metals and toxins below bioavailability [22]. It might bind histamine very well, as it is a relatively universal adsorber by nature. Hence, even when DAO tests show inconclusive results, PCT could be applied and probably prevent allergic reactions due to histamine binding.

The use of various antibodies against IL-5, IL-4, and IL-13 to block the eosinophilic pathway did not result in any clear clinical improvement. In a mouse model, the inhibition of the alarmins IL-25, IL-33, and TSLP was effective in preventing food allergies [2].

In the Codex Alimentarius, which has existed for more than 40 years, and the European regulation of gluten [9] in food, there are well-defined limits: 20 ppm or below means “gluten-free”, whereas <100 ppm corresponds to “very low gluten”. Food containing gluten at non-detectable levels or levels up to 20 ppm are considered safe for consumption by patients with celiac disease [9,11]. However, reports have found that even small amounts of only 50 mg/day might damage the intestinal mucosa. Like peanuts, gluten can contaminate food when the same production facilities are used or during harvesting, storage, transportation, etc., but there are also hidden sources, as gluten is used as an additive, for example, as a thickener, flavor enhancer, emulsifier, filler, and fortification ingredient [57]. Just like celiac disease, IgE-mediated wheat allergy, and nonceliac gluten sensitivity, there is no cure, and the avoidance of gluten in one’s diet is the only means of treatment. Proteases of the digestive system (such as trypsin, pepsin, and chymotrypsin) can only partially hydrolyze gluten, as the cleavage sites are largely restricted to regions that are immunologically silent [5]. This protease resistance is similar to that found in some Ara h proteins. Therefore, a sorption agent that can bind prolamins in general is needed to prevent both allergy induction and cross-reaction. It was previously found that, depending on the gluten source, 80–130 µg of gluten per 1 mg of PCT was bound, which corresponds to a binding capacity of 2 mg of gluten per 1 g of PCT [26]. Now, the following question can be addressed: can gluten and peanut protein bind simultaneously, or is one allergen adsorbed to PCT with a higher affinity than the other? Experiments were performed on the concomitant adsorption of peanut and gluten in a ratio of 1:1 (150 µg/mL each) to PCT at a dose of 1 mg/mL, and analyses were carried out using an ELISA specific to either peanut protein or gluten. As controls, the single peanut or gluten proteins were used in an individual approach. In the case of gluten extracted from wheat flour, more than 90 µg/mL was sorbed, and about 60 µg/mL remained free in the suspension, whereas PCT bound to proteins derived from peanut mush at an amount of more than 130 µg/mL, leaving 20 µg/mL free in the suspension. When using the gluten–peanut protein mix solution (again, 150 µg/mL each), 50 µg/mL of gluten and 70 mg/mL of peanut protein were sorbed to PCT. Considering the individual binding affinity of gluten to PCT in relation to that of peanut to PCT, the relationship is about 1:1.7, while it is 1:1.4 when regarding the affinities in the mix solution. Taking into account the naturally occurring fluctuations in experimental results, it was clear that both allergens bound with nearly the same affinity to PCT under competing conditions, thereby preventing their bioavailability (Table 2 and Figure 9).

Purified clinoptilolite-tuff, which was used for all the experiments performed and presented in this publication, is a member of the natural zeolite family. Native clinoptilolite itself has been used in recent times for human application, but it has a decades-long history as a feed additive for farm animals and pets [15,58]. A pioneer of zeolite administration is Japan, where, as far back as 1965, the first specific tests of using zeolite as a dietary supplement in ruminants, swine, and poultry were performed [16,59]. In the European Union, clinoptilolite-tuff is allowed for use as a feed additive [15,30,58,60]. In the United States of America, the raw material of PCT, clinoptilolite of sedimentary origin, is generally recognized as safe “for use as an anticaking agent in diets for cattle, swine, goats, sheep, poultry, cats, and dogs at a level up to 1 % by weight in complete diets” [61]. The presence of mycotoxins in human food and animal feed is a relevant problem; in particular, environmental determinants can promote rising concentrations and hence lead to both physical danger and food loss, as an estimated 30–50% of food commodities are lost pre- to post-harvest globally per year [62]. Mycotoxicosis is a severe risk for livestock, and mycotoxin binders help to overcome the acute or chronic contamination of animals by sorbing highly poisonous fungal toxins. Zeolites are known to be suitable mycotoxin adsorbers [63]. As peanuts are often contaminated with mycotoxins, the use of PCT against allergenic reactions could probably also tackle the problem of fungal toxin poisoning.

Zeolites became well known for their ad-/ab-sorbing capacities because of both their ion exchanging ability and the binding capability of the surface of their crystal lattice. On this basis, research has expanded on PCT. It is now known that purified clinoptilolite-tuff can render harmful toxins harmless by binding them, thereby preventing uptake into the (human) body [18,19,24,25,26,27]. Another documented quality of PCT is its ability to promote wound repair and regeneration [20]. With the present study, we add new information to the field of application of PCT, as we demonstrate that purified and therefore safe clinoptilolite-tuff in vitro can irreversibly bind the peanut matrix in relevant amounts, even when gluten proteins are present at the same time. A prospective human study could prove that the effect of PCT helps persons with peanut allergy in their everyday lives.

## 4. Materials and Methods

The laboratory equipment used is GLP-qualified.

### 4.1. The Chemical Compositions of Buffers Used in the Experiments Performed

Buffers of different kinds were used for various experiments. Table 3 provides an overview of the specific buffers, including their single components, concentrations, and pH values (according to the European Pharmacopoeia [30]).

### 4.2. Artificial Fluid Preparation

Both artificial fluids were prepared according to the recommendations of the European Pharmacopoeia [30].

#### 4.2.1. Preparation of Artificial Gastric Fluid

Pepsin (P7000, Sigma-Aldrich, St. Louis, MO, USA), in an amount of 3.2 g, was dissolved in 200 mL of ultrapure water prior to being inactivated via autoclavation (Certoclav Tisch-Autoklav, CV-EL 12L/18L, Sterilizer GmbH, Traun, Austria) at a maximum temperature of 120 °C for 25 min (including the heating-up time). Then, the pressure inside the autoclave was reduced slowly by hand, and, subsequently, the temperature of the solution was reduced to room temperature under constant stirring (Heidolph MR 3001 Heated Magnetic Stirrer, Schwabach, Germany). This deactivation step was crucial for performing ELISA testing, as previous experiments revealed that functional proteolytic pepsin degrades not only peanut protein but also the antibodies provided in the ELISA kit, rendering accurate measurement impossible. Afterwards, 2.0 g of NaCl (sodium chloride, 71380, Sigma-Aldrich, St. Louis, MO, USA) was added and dissolved before 80 mL of HCl [1 M] (hydrochloric acid 25%, CVH Chemie-Vertrieb GmbH & Co, Hannover, Germany) was pipetted into the solution. Finally, ultrapure water was added to reach a final volume of 1000 mL. The measured pH value was 1.19 at room temperature.

#### 4.2.2. Preparation of Artificial Intestinal Fluid

Pancreatin (P1750, Sigma-Aldrich, St. Louis, MO, USA) in an amount of 10 g was dissolved in 200 mL of ultrapure water before being inactivated via autoclavation at a maximum temperature of 120 °C for 25 min (including the heating-up time). As described for the preparation of the artificial gastric fluid, this step was important to ensure reliable ELISA analyses. Next, 250 mL of KH_2_PO_4_ [0.2 M] was mixed with 77 mL of NaOH [0.2 M] (sodium hydroxide solution, ≥32%, extra pure, Carl Roth, Karlsruhe, Germany) and added to the dissolved pancreatin solution. Then, the pH was fixed to 6.8, and ultrapure water was used to obtain a final volume of 1000 mL.

### 4.3. The Extraction of Proteins Derived from Either Peanut Mush or Wheat Flour

The extraction of proteins from roasted peanut butter (PB2 powdered peanut butter—the original, Bell Plantation Foods Inc., Tifton, GA, USA, 1121256130, best by 06/06/24), unroasted peanut mush (Erdnussmus aus rohen Erdnüssen, Ingvie, Wiggensbach, Germany, Batch 121222, Mindesthaltbarkeitsdatum 06 2025), and wheat flour (Österreichisches Weizenmehl, griffig, naturrein, Type 480, Spar, Salzburg, Österreich, L427, 02/21:41, 10/2024) for analysis using an ELISA and Coomassie blue reagent was carried out as detailed below.

#### 4.3.1. Extraction of Peanut Proteins

The peanut butter or mush extract was used for all in vitro experiments, except for the digestion of peanut products in artificial gastrointestinal fluids, where imitation of in vivo digestion was the aim. Then, the unmodified peanut foodstuff was directly used without any prior preparation.

First, 2 × 1 g of each peanut product was mixed with 3 mL of n-hexane (Merck, Darmstadt, Germany) using a rotator (40 rpm, rotator SB3, Stuart, Staffordshire, UK) for 60 min. Afterwards, both samples were kept in a closed tube overnight to generate a pellet via gravitation. The supernatant was discarded the next day, and the defatting process was repeated 3 times in total. Then, the two pellets were dried over 3 days in a fume hood (Filterabzug mc6, Waldner Laboreinrichtungen, Wangen im Allgäu, Germany) at room temperature. Both pellets were pooled, mixed with 30 mL of 1 M TRIS-HCl buffer (pH 8.5, TRIS Hydrochloride > 99.5%, Apollo Scientific, Bredbury, Stockport, UK), and mixed well under rotation (40 rpm) for 30 min before centrifugation at 12,600× *g* (Biofuge primoR, Heraeus, Hanau, Germany) for 5 min. The supernatant was mixed vigorously (Vortex Mixer SA8, Stuart, Staffordshire, UK) and kept at −80 °C (Forma 900 Series 5905, Thermo Fisher Scientific, Marietta, OH, USA) in small aliquots of 510 µL each until use.

#### 4.3.2. Extraction of Wheat Proteins

In 2 reaction tubes, 1 g of each tube of wheat flour was weighed (Sartorius CP225D, Göttingen, Germany) and mixed with 3 mL of n-hexane. A suspension was generated via rotation (17 rpm) at room temperature for 60 min. For the generation of a pellet, the suspension was kept in closed tubes under a fume hood for 30 min. After the removal of the formed supernatant, again, 3 mL was added to each tube of n-hexane, and the defatting process described above was repeated another two times before the drying of the pellet (with the cap of the tube removed) under the fume hood at room temperature overnight. The pellets were merged and mixed (17 rpm) with 20 mL of 1 M TRIS-HCl buffer (pH 8.5) at room temperature for 30 min. The suspension was centrifuged at 12,600× *g* for 5 min before collecting the resulting supernatants and mixing them. Finally, samples of the supernatants were frozen in small portions of 510 µL each at −80 °C prior to their use in experiments.

### 4.4. Preparation of the PCT Suspension

For the preparation of the PCT (G-PUR^®^, Glock Health, Science and Research G.m.b.H., Deutsch-Wagram, Austria, Lot. 037-03-08-4-0-0) suspension, 500 mg of purified clinoptilolite-tuff was mixed vigorously with 10 mL of ultrapure water and sonicated (Sonorex RK 103 H, Bandelin, Berlin, Germany) at room temperature for 15 min. Immediately prior to its use, the suspension was well vortexed again.

### 4.5. Adsorption and Desorption of Peanut Proteins and Gluten from Purified Clinoptilolite-Tuff

The binding of allergens to PCT is crucial for PCT’s mode of action to isolate and inactivate them to prevent negative effects in the digestive system. Basic in vitro research experiments should characterize the optimal relative quantities as well as the best incubation time and milieu between the adsorber and peanut proteins.

Subsequently, it was determined that in vivo-like tests should give a more detailed insight into the mechanism of allergen binding. Therefore, conditions aiming to simulate the human digestive process were created by using artificial gastric and intestinal fluids.

Consequently, the question was raised as to whether the bond between the allergen and PCT is strong enough to pass through the digestive tract or whether it is only of a temporary nature. So, desorption experiments were also performed in the artificial fluids.

To simulate gluten-contaminated food and test whether there binding still occurred between PCT and peanut proteins, gluten from wheat—known to have a strong affinity for PCT [26]—was used as a competing factor for peanut protein adsorption.

#### 4.5.1. The Generation of a Saturation Curve

To characterize the adsorption of peanut protein to PCT, the peanut protein extract was used at various concentrations (0 µg/mL, 6.25 µg/mL, 12.5 µg/mL, 25 µg/mL, 50 µg/mL, and 100 µg/mL) and at 2 different pH values (pH 1.5 and pH 6.8).

The total protein content of the single samples was analyzed using Coomassie blue reagent (Coomassie (Bradford) Protein Reagent, Thermo Scientific, Rockford, IL, USA) and an ELISA (R6811, R-Biopharm AG, Germany) to determine whether similar results could be obtained using both methods. For the experiments analyzed using the Coomassie reagent and ELISA, either raw peanut mush or extract was used.

A concentration of 1 mg/mL of PCT was used.

The tests were carried out at 37 °C to imitate human body temperature. All test solutions used were pre-warmed in a water bath (Medingen WB 10, P-D Industriegesellschaft mbH Prüfgerätewerk Dresden, Dresden, Germany). The incubation for adsorption testing using a rotator was carried out in an incubator (KBF 115, Binder, Tuttlingen, Germany) at 37 °C.

In the case of peanut mush, 100 mg of unroasted raw material was weighed and mixed with pre-warmed (37 °C) pH 6.8 buffer or pH 1.5 test solution for 15 min. Afterwards, the suspension was centrifuged (MEGA STAR 1.6R, VWR, Radnor, PA, USA) at 4100× *g* for 10 min at room temperature before the supernatant was filtered through filter paper (Whatman quantitative filter paper, ashless, Grade 42, Schleicher & Schuell, Buckinghamshire, UK) to separate insoluble parts from the solution. Then, the filtrate was warmed up again to 37 °C for 20 min before the adsorption process was carried out.

Otherwise, when the extract was the source of peanut protein, no prior treatment was necessary, and the extract was used for the experiment directly.

For the experiment, either filtrate or extract (in different concentrations) was used and merged with PCT. The suspension was incubated at 37 °C with rotation (17 rpm) for 30 min and then centrifuged at 4100× *g* at room temperature for 10 min. Then, analyses with the ELISA and Coomassie reagent were performed immediately in parallel so that the factor of time did not need to be accounted for concerning the results of both methods.

#### 4.5.2. The Influence of pH on Peanut Protein Binding

We determined whether there was an influence of acidity or alkalinity on peanut protein adsorption to purified clinoptilolite-tuff by incubating peanut protein together with 1 mg/mL of PCT for 30 min at 37 °C under rotation (17 rpm) in different buffers, including pH 7.8, 6.8, 5.8, and 4.5 buffers, and a solution of pH 1.5 (Table 3). Then, the samples were centrifuged at 4100× *g* for 10 min, and the supernatants were analyzed using an ELISA (R6811), according to the manufacturer’s guidelines. OD measurements were taken at a wavelength of λ = 450 nm by using a plate reader (Biotek synergy HT, Winooski, VT, USA). Based on these data, the adsorption capacities were calculated.

#### 4.5.3. The Kinetics of Peanut Protein Adsorption to PCT

To characterize the binding of peanut proteins to PCT in a time-dependent manner, an ELISA (R6811) was used. The extracted peanut proteins were diluted in phosphate pH 6.8 buffer containing 250 mL of 0.2 M KH_2_PO_4_ (potassium dihydrogen phosphate, 26931.263, VWR, Radnor, PA, USA) and 112 mL of 0.2 M NaOH in a final volume of 1000 mL and a final concentration of 50 µg/mL. Purified clinoptilolite-tuff was diluted in the same buffer to yield a final concentration of 2 mg/mL. Experiments involving controls containing only peanut proteins in the suspension and samples with PCT added were each performed in quadruplet. All samples were incubated at 37 °C under rotation (17 rpm) for time periods of 1 min to 30 min. The samples were centrifuged at 4100× *g* for 10 min at room temperature prior to determination of the content of peanut proteins in the supernatants using an ELISA, following the manufacturer’s instructions. Measurements were performed at a wavelength of λ = 450 nm by using a plate reader.

#### 4.5.4. Adsorption Mimicking in vivo Conditions by Using Artificial Gastric and Intestinal Fluids

In the experiment with artificial gastric fluid, unroasted raw material at a final concentration of 45 µg/mL of whole peanut was weighed and mixed with 150 mL of pre-warmed (37 °C) artificial gastric fluid via magnetic stirring for 15 min. Afterwards, the peanut protein suspension in the artificial gastric fluid was mixed with PCT and incubated with rotation (17 rpm) at 37 °C for 30 min before centrifugation at 4100× *g* at room temperature for 10 min. The resulting supernatant was used immediately for ELISA analyses. The single approaches included a control and 0.5 mg/mL, 1 mg/mL, and 2 mg/mL of PCT.

All the tests were performed in quadruplet.

For the testing of PCT binding to peanut components in the artificial intestinal fluid, unroasted raw material at a final concentration of 100 µg/mL of whole peanut was weighed and mixed with 100 mL of pre-warmed (37 °C) artificial intestinal fluid via magnetic stirring for 15 min. Afterwards, the solution was filtered through filter paper (Grade 42) to separate insoluble parts from the solution. Then, 2.6 mL of the filtrate was diluted with 97.4 mL of warmed-up (37 °C) artificial intestinal fluid before the adsorption process was carried out. As carried out with the artificial gastric fluid, PCT was mixed with the artificial intestinal fluid and incubated on a rotator at 17 rpm and 37 °C for 30 min. Afterwards, PCT was separated from the solution via centrifugation at 4100× *g* for 10 min at room temperature. Again, the supernatant was analyzed using an ELISA. The individual approaches included a control and treatments with 0.25 mg/mL, 0.5 mg/mL, and 1 mg/mL of PCT.

All tests were performed in quadruplet.

#### 4.5.5. Desorption of Peanut Proteins from Purified Clinoptilolite-Tuff

To prove the strength of the binding between the peanut protein allergen and PCT and ensure constant allergen adsorption during passage through the digestive tract in an in vitro model, tests of the following processes and conditions were carried out:

(a) Adsorption in the artificial gastric fluid and desorption in the gastric fluid or pH 1.5 test solution;
(b) Adsorption in the artificial gastric fluid and desorption in the intestinal fluid or pH 6.8 buffer;
(c) Adsorption in the artificial intestinal fluid and desorption in the intestinal fluid or pH 6.8 buffer.

All desorption experiments were initially performed with corresponding pH 1.5 test solution (for stomach) and pH 6.8 buffer (intestine) before changing to artificial fluids. Both the artificial fluids and buffer/test solution yielded similar results.

First, the previously described adsorption was performed with either the artificial gastric or intestinal fluid and unroasted raw peanut mush. Then, the supernatants obtained through centrifugation were kept for analysis using a peanut ELISA. For desorption, the resulting pellet was resuspended in either the buffer/test solution or artificial fluid (all tempered at 37 °C) and incubated at 37 °C with rotation (17 rpm) for 10 min prior to centrifugation at 4100× *g* and room temperature for 10 min. In the following, incubation in the buffer/test solution/artificial fluid took place for 5 min under the conditions previously described. This step was repeated another 2 times. All supernatants created either by adsorption or desorption were used in an ELISA.

All tests were performed in quadruplet.

The PCT concentration for adsorption in the gastric fluid was 1 mg/mL, and, in the intestinal fluid, it was 250 µg/mL.

#### 4.5.6. Competing Adsorption in Buffers and Test Solutions between Peanut Proteins and Gluten from Wheat

Simultaneous adsorption was achieved by using the same concentrations of peanut protein and wheat gluten (170 µg/mL each). Three different approaches in pre-heated (37 °C) pH 6.8 buffer were applied:

(a) A mixture of peanut protein and wheat gluten in a ratio of 1:1;
(b) Peanut protein alone;
(c) Wheat gluten alone.

PCT was used at a concentration of 1 mg/mL. All tests were performed in quadruplet. Incubation took place at 37 °C in a rotator (17 rpm) for 30 min. PCT was separated from the extracts via centrifugation at 4100× *g* and room temperature for 10 min.

Peanut and gluten ELISAs were performed immediately afterwards, simultaneously using the supernatants of either the mixture and peanut protein or the mixture and wheat gluten, respectively.

### 4.6. The Determination of Protein Concentrations with Coomassie Blue Reagent

The total protein content of the single samples was analyzed using Coomassie blue reagent.

Therefore, a standard curve composed of different bovine serum albumin (BSA, Albumin Standard, Thermo Scientific, Rockford, IL, USA) concentrations (0 µg/mL ≙ blank, 2.5 µg/mL, 5 µg/mL, 10 µg/mL, 15 µg/mL, 20 µg/mL, and 25 µg/mL) was prepared in either ultrapure water or buffers with specific pH values.

The individual samples were diluted 1:20 in buffers or test solution with corresponding pH values.

After using either 150 µL of the prepared standards or diluted samples in duplicate in a 96-well plate, both were mixed 1:1 ^V^/_V_ with Coomassie blue reagent. Then, the plate was rocked gently, and the protein content was measured using a spectrophotometer at a wavelength of λ = 595 nm.

The experiments were performed at least 3 times per approach.

### 4.7. Determination of Peanut Protein Content via ELISA

Peanut proteins were detected by using a RIDASCREEN Peanut ELISA kit (R6811), which is a sandwich enzyme immunoassay validated for the quantitative analysis of peanut and peanut protein in food. It is certified by AOAC-RI (PTM No. 112102) and has a limit of detection of 0.15 mg/kg (ppm) for whole peanut (corresponding to 0.033 mg/kg (ppm) of peanut protein) and a limit of quantification of 0.75 mg/kg (ppm) for whole peanut (corresponding to 0.166 mg/kg (ppm) of peanut protein).

The content of peanut proteins in the supernatants was determined using a RIDASCREEN Peanut ELISA kit (R6811) according to the manufacturer’s instructions before measurement of the samples with a spectrophotometer at a wavelength of λ = 450 nm.

### 4.8. Determination of Gluten Content via ELISA

Gluten content was determined by using a RIDASCREEN Gliadin ELISA kit (R7001, R-Biopharm AG, Germany), which is especially designed for the quantitative analysis of wheat, rye, and barley prolamins in food declared to be gluten-free. It is certified as a Codex Alimentarius Method (Type I) [9] and by the AOAC (PTM No. 120601). It has a limit of detection of 0.5 mg/kg of gliadin (≙ 1.0 mg/kg gluten) and a limit of quantification of 2.5 mg/kg of gliadin (≙ 5.0 mg/kg gluten). Prolamins react with the monoclonal R5 antibody provided in the kit.

The gliadin content of the supernatants was determined using a RIDASCREEN Gliadin ELISA kit (R7001) before the measurement of the samples with a spectrophotometer at a wavelength of λ = 450 nm.

It is important to note that the content of gluten corresponds to twice the amount determined for gliadin/hordein/secalin. This is based on the assumption that the prolamin/glutelin ratio is 1:1.

## 5. Conclusions

Due to its severe allergic effects, peanut allergy can be life-threatening. For affected persons, this is problematic, as peanuts are widely used for various applications in the food industry. The declaration of unintended peanut traces is optional, and cross-reactions with other allergens are common. Although several treatment options have been tested, the only means of treatment for allergic shock is injectable epinephrine. As the prevalence of peanut allergy is rapidly increasing, other medical options are needed.

In this paper, the results of in vitro studies, using purified clinoptilolite-tuff and the whole peanut matrix, as well as isolated peanut protein, are shown. We demonstrated that PCT is capable of sorbing peanut protein in reasonable amounts under conditions mimicking the human body (37 °C and the pH of the stomach and intestine). Artificial fluids of both organs were used for further investigations under conditions simulating human digestion. Finally, focus was placed on the capacity of PCT to adsorb two allergens at once—peanut and gluten proteins. Analyses were performed using an ELISA (peanut and gluten, respectively) and a Coomassie (Bradford) assay.

The reaction kinetics were fast. Within the first 2 min, approximately 65% of the peanut protein had adsorbed to PCT at pH 6.8. The results indicate the pH dependency of the adsorption capacity: whilst 40 µg of peanut protein was bound to 1 mg of PCT, only half of this amount was sorbed under acidic conditions at pH 1.5. This was also true for the experiments in which artificial fluids were used. Desorption testing revealed irreversible binding adsorption under gastric conditions and only traces of free protein at intestinal pH, leading to the assumption that, under the tested conditions, peanut allergens were bound in the stomach and thus could not induce an immune reaction in the intestine. The concomitant binding of peanut and gluten proteins demonstrated that both allergens were adsorbed at the same sites with equal affinity.

## Figures and Tables

**Table 2:** Concomitant sorption of gluten and peanut to PCT in comparison to binding of the single proteins to PCT. Data from one representative experiment are shown.

Test Approach	Protein Sorbed to PCT [µg/mg]	SD [µg/mg]
Peanut protein only	136.5	13.8
Peanut protein in mix	63.1	14.1
Gluten only	91.0	18.1
Gluten in mix	48.1	14.3

**Table 3:** Preparation of phosphate buffers and test solution. Single buffers were prepared from chemicals with the highest purification grade. Ultrapure water (created by Sartorius arium mini, Göttingen, Germany) was used as solvent. pH values were fixed via the addition of either 0.1 M NaOH solution or 0.1 M HCl solution. pH measurement was performed by using a precision pH meter (pH 3210i, WTW, Xylem Analytics Germany Sales GmbH & Co. KG, Weilheim, Germany) in combination with a suitable pH electrode (Sentix 81, WTW, Xylem Analytics Germany Sales GmbH & Co. KG, Weilheim, Germany) featuring an integrated NTC temperature sensor at room temperature.

Phosphate Buffer [pH]	KH_2_PO_4_ Solution [0.2 M] Volume [mL]	NaOH Solution [0.2 M] Volume [mL]	Final Volume [mL]
6.8	250	112	1000
5.5	250	12	1000
**Phosphate Buffer** **[pH]**	**KH_2_PO_4_** **[g]**		**Final** **Volume** **[mL]**
7.5	44.91		1000
4.5	13.61		1000
**Test Solution** **[pH]**	**NaCl Solution** **[0.2 M]** **Volume [mL]**	**HCl Solution** **[0.2 M]** **Volume [mL]**	**Final** **Volume** **[mL]**
1.5	250	207	1000

## Data Availability

All data generated and analyzed are included in the published article.
